# Choriocarcinoma: Diagnosis, treatment and management of a rare germ cell tumour. An update review

**DOI:** 10.1177/03008916251408266

**Published:** 2026-02-23

**Authors:** Pierantoni Francesco, Fabiana Caliciotti, Selma Ahcene Djaballah, Eleonora Lai, Davide Bimbatti, Silvia Stragliotto, Ilaria Zampiva, Melissa Ballestrin, Chiara Pittarello, Salim Jubran, Greta Pretto, Elisa Erbetta, Anna Milani, Umberto Basso, Marco Maruzzo

**Affiliations:** 1 Oncology 3 Unit, Istituto Oncologico Veneto, IOV – IRCCS, Padova, Italy; 2 Department of Surgery, Oncology and Gastroenterology, University of Padua, Padova, Italy; 3 Oncology 1 Unit, Istituto Oncologico Veneto, IOV – IRCCS, Padova, Italy

**Keywords:** germ cell tumours, choriocarcinoma, GCT, NSGCT, non-seminomatous germ cell tumours

## Abstract

Choriocarcinoma is a malignant neoplasia which develops from trophoblastic cells. In males it is rare and often associated with other non-seminomatous germ cell tumours of the testis. Choriocarcinoma often presents with metastatic disease and elevated βHCG levels. Usually, patients' symptoms are associated with the different metastatic sites and they can be severe or even life-threatening. Moreover, choriocarcinoma is chemosensitive and the administration of chemotherapy with curative intent may lead to tumour-lysis syndrome and the more specific choriocarcinoma syndrome (CS). Therefore, the treatment of metastatic choriocarcinoma is complex, involving both oncological therapy and the management of acute complications. This review explores choriocarcinoma in males, focusing on its clinical presentation, pathogenetic mechanisms, and treatment options, including investigational therapies. Additionally, we aim to highlight the severe complications of CS and discuss its management strategies.

## Introduction

Testicular neoplasms can be divided into germ cell tumours (GCTs), stromal tumours and others (e.g., testicular lymphomas). GCTs account for almost all testicular cancers.

GCTs can be classified as seminomatous germ cell tumours (SGCTs) or non-seminomatous germ cell tumours (NSGCTs).

GCTs are relatively rare tumours and are the most common type of malignant disorder in adolescents and young adults (AYAs; between 15 and 39 years old), with nearly 74,500 new cases estimated globally in 2020.^1^ Although the incidence of testicular germ cell tumour (TGCT) has increased sharply in recent years, GCT incidence rates are likely to increase even more, particularly in geographical areas with lower incidences (e.g. Eastern Europe). This increase may be due to exposure to endocrine-disrupting chemicals, although the etiology of GCTs remains uncertain.^2,3^ TGCTs have been associated with cryptorchidism, hypospadias, Klinefelter syndrome and decreased fertility — the so-called testicular dysgenesis syndrome.^4^ Contralateral TGCT and germ cell neoplasia in situ (GCNIS) are also important risk factors, as is familiarity, even though no associated genes have been identified to date.^5^ The majority of seminomas (85%) are diagnosed as stage I, probably due to a slower growth rate. On the other hand, only 60% of non-seminomas present as stage I disease.

The vast majority of GCTs occur in the testicles (about 95%), while only 5% develop outside the gonads. These entities are known as extragonadal GCTs (EGGCTs), and they are usually localised in the body’s midline (especially in the retroperitoneum and mediastinum).^6^ Overall, they represent one of the most curable malignancies and a model of curable solid cancer for their unique chemosensitivity. In fact, more than 95% of patients with GCTs achieve a 5-year survival^7^; Curability is approximately 99% in the first and second clinical stages, dropping to 75-80% in advanced stages.^8^

As far as the advanced stage is concerned, the International Germ Cell Cancer Collaborative Group (IGCCCG) has developed a clinically based prognostic classification. Patients are divided into subgroups of good, intermediate, and poor prognosis based on three main criteria: the primary tumour site (gonadal vs extragonadal), the levels of serum tumour markers, and the presence of extra-pulmonary visceral metastases. However, within the subgroup of poor-risk NSGCTs, a particular subset can be identified, known as ‘super-high-risk’ patients. This subgroup is characterised by the presence of pure choriocarcinoma at histological examination, with possible widespread lung metastases, and a high level of choriogonadotropin.

Choriocarcinoma is a rare subtype of NSGCTs and is the most aggressive due to its biological features. It has a high malignant potential and early hematogenous metastases, with about 30% of patients being diagnosed already in the metastatic stage.^7^ Choriocarcinoma is often associated with haemorrhagic complications, which can be life-threatening.^9^ The high proliferation rate and capability for vascular invasion favour choriocarcinoma hematogenous spread to the lungs, liver, and brain at an early stage. Indeed, patients usually present symptoms related to the metastatic sites, rather than a swelling testicular mass.

Additionally, choriocarcinoma is the GCT that most frequently shows histological regression, or ‘burned-out tumour’. Indeed, regression is less frequent in testicular seminoma and it is believed to not occur in teratoma, while it is possible in embryonal carcinoma.^10^

Another important feature of choriocarcinoma is its syndrome (CS). Patients with very advanced disease can develop CS, which presents with acute respiratory failure shortly after beginning systemic chemotherapy. This pathognomonic syndrome is worthy of more in-depth exploration of its etiopathogenesis, clinical manifestation, and how to identify high-risk patients for a better therapeutic approach, with the aim of reducing mortality in the early phases of systemic therapy without compromising the chances of long-term survival.^7^

## Choriocarcinoma’s clinical features

The clinical presentation of choriocarcinoma varies depending on the metastatic sites involved. Gastrointestinal bleeding and haemoptysis secondary to gastrointestinal tract and pulmonary metastases are possible choriocarcinoma presentations. Other symptoms may include back pain due to retroperitoneal lymphadenopathies and neurological symptoms from brain lesions.^11^

Another clinical feature associated with choriocarcinoma is a dramatic elevation of serum choriogonadotropin (βHCG).^12^

Indeed, high levels of βHCG can cause paraneoplastic syndromes such as hyperthyroidism, and gynecomastia. An elevated βHCG may cause hyperthyroidism because of a cross-reaction with thyrotropin stimulating hormone (TSH). Moreover, the increase in βhCG, being it a luteinising hormone (LH) analogue, may stimulate Leydig cells to produce higher oestrogen levels and lower testosterone, thus justifying the gynecomastia and atrophic testis which can be detected in some cases. Almost 1% of testicular cancer patients present gynecomastia.^13^

It should be remembered that drastically elevated β-hCG levels are also a poor prognostic factor in the IGCCCG classification.^14^

Pulmonary lesions are frequent and may be symptomatic, especially with haemorrhagic manifestations such as haemoptysis and, less frequently, dyspnoea. Considering diagnostic imaging, lung lesions usually show themselves as so-called ‘cannonball metastases’, which typically appear as multiple round well-defined pulmonary nodules.^15^

Choriocarcinoma frequently metastasises to the brain. The optimal treatment strategies of brain metastases remain controversial. Patients with brain lesions have a worse prognosis, but long-term survival can still be achieved with traditional platinum based chemotherapy and radiotherapy. Indeed, choriocarcinoma brain metastases are both radiosensitive and chemo-sensitive, and the disease usually affects young men with a good performance status, who can be treated aggressively. In case of large brain metastases (diameter > 3 cm) or single brain lesion neurosurgery should be considered, in the context of a multimodal treatment which also includes systemic chemotherapy and, in selected cases, radiotherapy after neurosurgery.^16,17^

Despite the best treatments, life-threatening adverse events such as intracranial haemorrhage of the metastases is a major cause of morbidity,^18^ and it can even be induced by chemotherapy. Given the high mortality associated with tumour haemorrhage, neurosurgery should be also considered a reliable option for preventing critical haemorrhage in the brain in this setting.^19^

Given that lower dose intensity of chemotherapy with cisplatin (BEP regimen) has been reported to result in worse survival rates, BEP should be initiated promptly, and with adequate doses.^20^ Whole brain radiotherapy (WBRT) is an option for treatment of brain metastases, but it carries the risk of neurotoxicity, and concurrent treatment with systemic chemotherapy may increase the rate of neurological long-term toxicities.^21,22^ However, in some retrospective studies neurotoxicity did not increase when etoposide and cisplatin were administered concurrently with cranial radiation.^23^ It should be noted that WBRT may be the only possible treatment in the case of widespread brain lesions.

Gastrointestinal involvement from choriocarcinoma metastases is possible, but unusual (less than 5% of cases): in the majority of cases the duodenum may be involved due to its anatomic contiguity to the retroperitoneal lymph nodes, which are the most common metastatic site for all GCTs. Small bowel, stomach, oesophagus, colon and pancreas are other sites which may be involved.^24^ Gastrointestinal metastases can manifest with melena, hematemesis, anaemia, intussusception, perforation, and pseudo-obstruction due to polypoidal growth, symptoms which may mimic more common gastrointestinal conditions such as appendicitis. Thus, choriocarcinoma should be considered in the differential diagnosis of causes of acute abdomen and gastrointestinal bleeding in young men.^25^

Testis is the most common primary site of choriocarcinoma, seen in 33.0% of cases. However, it can be of extragonadal origin. Primary extragonadal choriocarcinoma may occur in the retroperitoneum, in the mediastinum, and, rarely, in the pineal body.^26^

Many theories have been proposed explaining the pathogenesis of these extragonadal choriocarcinoma, but no conclusions have yet been reached. At the time of writing, there are three main hypotheses: the tumour is a metastasis of a choriocarcinoma (CC) in the testis that regressed spontaneously^27^; the tumour originated from the primordial germ cells that migrated during embryonic development^28^; and the tumour developed originally as a non-trophoblastic neoplasm and then it transformed into a CC.^29^

## Burned-out choriocarcinoma

The term ‘burned-out testicular tumour’ refers to partial or complete histological regression of the tumour in the testis associated with metastatic disease.^10^ Choriocarcinoma is the most frequent GCT subtype that shows this phenomenon, followed by embryonal carcinoma.

Burned-out testicular tumour is still a rare clinical entity, but it may complicate the diagnosis because patients may present with widespread metastases and no primary tumour except for an area of calcification within the testis.

The mechanism behind burn out has not yet been determined. This phenomenon could be explained by a high metabolic rate and rapid proliferation, which cause an ischemic response in the primary neoplasia; supporting this hypothesis, in most specimens of burned-out tumours it has been demonstrated both a lymphoplasmacytic infiltrate and the presence of hemosiderin-containing macrophages.^30^

Other histological features of this particular clinical finding are scar formation, intratubular calcifications and testicular atrophy.^31^ In a scrotal ultrasound, a burned-out tumour can show itself with intratesticular microlithiasis or microcalcifications. It should be noted that the prognosis of metastatic disease associated with burned-out tumour of the testis does not differ from regular metastatic testicular malignancy.^32^

## Choriocarcinoma syndrome

Choriocarcinoma syndrome (CS) is a specific clinical manifestation of choriocarcinoma. It has been described as a haemorrhagic syndrome with blood loss from metastatic sites in patients with TGCTs, associated with high βHCG levels. It usually occurs in the early phase of chemotherapy, with lung haemorrhage and acute respiratory failure, with a high mortality rate. While this is the most typical presentation, any metastatic site other than the lung may cause acute bleeding. The incidence is not well-defined. CS has also been described in non-pure choriocarcinomas; in the literature some cases of mixed GCTs with a minor portion of choriocarcinoma have been described.^7^ CS is caused by massive tumour cell lysis as a result of chemotherapy and a consequent cytokine release. The local cytokine storm in the lung then causes alveolar haemorrhage, and thus acute respiratory failure. Consequently, the patient is in a frail condition, which often leads to a super-infection, fostered by chemotherapy related immunosuppression and neutropenia. The clinical situation may also worsen even more with a systemic inflammatory response, and multiorgan failure. Indeed internal bleeding, and thus CS, has been implicated as the cause of death in 44% of men with metastatic choriocarcinoma at autopsy. CC cells have the capability of directly invading and destroying blood vessels, but the pathogenesis of the intra-alveolar haemorrhage is probably to be looked for in tumour-lysis, with its release of substances such as uric acid, potassium and cytokines. As already stated, to improve survival chances for patients with metastatic choriocarcinoma it is of paramount importance to start chemotherapy as soon as possible, and with an adequate dose. Consequently, the prevention and treatment of choriocarcinoma syndrome could be tricky and will be described in detail in the next section.

## Diagnosis and treatment

In the context of GCTs which are considered highly curable diseases, testicular choriocarcinoma is characterised by a higher probability of developing cisplatin-refractory or progressive disease. Failure to achieve complete tumour remission after first line chemotherapy is indeed an indicator of poor prognosis.

In the most frequent CC clinical presentation, which is metastatic disease, radical orchiectomy should nevertheless be always performed, allowing for proper histopathological diagnosis and control of the primary neoplastic mass, considering the presence of the blood-testis barrier. A CT (computed tomography) scan is mandatory to determine the stage of the disease, while other imaging methods, such as positron emission tomography (PET) scans, are usually not used to stage NSGCTs due to the possibility of false negatives.^33^ In highly selective cases of metastatic germ cell tumours (GCTs) with poor prognosis, where chemotherapy must be initiated as soon as possible, it may be necessary to postpone orchiectomy until after the first chemotherapy cycle or at the end of the chemotherapy regimen. It is very important not to miss the chance to initiate therapy. For this reason, treatment is sometimes started based on a clinical diagnosis based on imaging and circulating tumour markers such as β-hCG while awaiting histological confirmation. From a therapeutic perspective, for retroperitoneal or mediastinal non-seminomatous germ cell tumours (NSGCTs), including CC, with elevated serum tumour markers, an upfront chemotherapy approach with four cycles of bleomycin, etoposide and cisplatin (BEP) is recommended, eventually followed by surgical reassessment. In the case of mediastinal NSGCTs, some authors suggest using the VIP regimen (ifosfamide instead of bleomycin in combination with cisplatin and etoposide) instead of BEP to avoid potential pulmonary complications induced by bleomycin.^33^

Due to their specificity and sensitivity of the tumour markers for GCTs (and in particular for CC), they are excellent tools for both for diagnosis and for assessing treatment response. Even lactate dehydrogenase (LDH) may be elevated in GCTs, and its level correlates with the extension of the metastatic burden, even if it is not as specific as β-hCG. Serum tumour markers are also used to correctly classify the disease in its risk class, as already described in the introduction, and then in the follow up phase.^34^ In the case of suspected CC, β-hCG should be determined before and after carrying out orchiectomy. Even with negative imaging, a persistent or increasing β-hCG after orchiectomy always indicates metastatic disease, and the necessity of BEP chemotherapy (CC stage IS).

In recent years miRNAs are emerging as possible circulating biomarkers for GCTs. These little RNA fragments are fairly stable in extracellular fluids and can be measured by assay, such as quantitative polymerase chain reaction (qPCR). The specificity of miRNA miR-371a-3p seems to be higher than the classical AFP, beta-HCG and LDH markers: indeed, more than 86% of GCTs patients express this miRNA.^34^

In case of a negative staging, patients with stage I CC or with a mixed non-seminoma with a CC component without lymphovascular invasion should be informed about the risk of overtreatment with adjuvant chemotherapy, and usually a follow-up plan is proposed. On the other hand, patients with vascular invasion have a higher relapse rate (40% to 50%). One course of adjuvant BEP should be considered the standard treatment in this setting. The relapse rate after one cycle of BEP is <5%.^35^

Semen analysis and sperm cryopreservation should be offered to all patients, if possible before orchiectomy, and it is mandatory before BEP chemotherapy, as they will receive treatment that has a serious risk of compromising fertility. The cumulative doses of cisplatin determine the extent of damage to spermatogenesis so BEP with curative intent has a higher risk of inducing infertility than adjuvant chemotherapy.^36^

For stage IIA non-seminoma with negative serum markers, enlarged lymph nodes may not contain metastases. The best strategies include close follow-up, lymph node biopsy, or primary nerve sparing (retroperitoneal lymph node dissection, RPLND), which has both diagnostic and therapeutic potential. If viable germ cell tumour (GCT) is found in the specimen, adjuvant chemotherapy post-RPLND should be considered. However, if the specimen consists entirely of pure teratoma, chemotherapy should not be administered. If serum markers are positive or stage IIb, the treatment should follow the metastatic disease approach, according to IGCCCG recommendations, three cycles or four cycles of BEP according to the classification of good or intermediate/poor prognostic groups.^36^

The prompt initiation of chemotherapy is also a well-known determinant of prognosis of metastatic choriocarcinoma. BEP or VIP (etoposide, ifosfamide, cisplatin) regimens are recommended for first-line therapy, with prophylactic G-CSF administration in order to reduce haematological side effects and avoid treatment delay, which is usually associated with a poorer outcome.^37^

Residual disease is common after chemotherapy. For non-seminoma residual masses with negative serum markers, surgical resection is recommended if larger than 1 centimetre.^33^

NSGCTs are relatively radioresistant compared to seminomas, and radiotherapy has been consequently excluded from curative strategies for these histotypes. However, modern techniques including stereotactic body radiotherapy (SBRT), which enables the delivery of very high radiation doses in small volumes, have made radiotherapy feasible in the context of a multimodal treatment. The potential applications of SBRT is in particular for the treatment of brain lesions, or for ablative therapy of residual masses after systemic treatment that cannot be surgically removed.^38^

Chemotherapy remains the main therapeutic weapon for treating stage II and III CC, but even and adequate systemic treatments may be complicated by CS. Mostly, the haemorrhagic symptoms appear shortly after the introduction of chemotherapy, but in some cases they can manifest with a pretreatment onset.^39^

Current data regarding optimal treatment approaches for CS are limited. Patient referrals to tertiary centres should be considered. As expected, current evidence suggests that the best results for these patients are obtained in high-volume reference institutions. Because of the rarity and complexity of this group of patients, it is recommended that they be referred to specialised centres as soon as possible (within 24h) to optimise survival chances. While the evidence is mainly from retrospective data, TGCT guidelines included this recommendation.^40,41^ Other studies suggest that the administration of chemotherapy should be started in an intensive care setting, to improve the treatment outcome and minimise the risks for the patient.^7^

A possible approach in extensive disease to reduce the risk of lysis and thus CS, could be a first shortened course of chemotherapy and/or a reduced dosage in induction chemotherapy before full dose. Massard et al.^42^ suggested a modified EP regimen of three days of etoposide-cisplatin (so without bleomycin), and the remaining two days of chemotherapy were postponed to day 15. After this first phase of induction, the full-dose BEP regimen with standard doses should start on day 21. In their experience this approach lowered the incidence of ARDS by 57% (87% vs. 30%) and the mortality rate by 40% (60% vs. 20%). In their report long-term survival showed an increase from 27% to 40%.^42^

These retrospective data underline the importance of treatment personalisation based on the metastatic burden and the risk of developing CS. Thus, induction with etoposide and cisplatin without bleomycin for patients with high-volume lung metastases, should be considered.^7^

For cases of refractory disease, in which β-hCG levels plateau or rise after chemotherapy, or there is a radiographic disease progression, salvage chemotherapy treatments should be considered.

About 20% of patients with CC will progress after first-line therapy: 11% of them within the IGCCCG low-risk group, 25% in the medium-risk group, and 46% in the high-risk group. The options for treating relapsed cases include salvage surgery, conventional-dose chemotherapy (CDCT), and high-dose chemotherapy (HDCT) followed by autologous blood stem cell transplantation.^43^ Lorch et al.^44^ studied conventional-dose versus high-dose chemotherapy as the first salvage treatment in male patients with metastatic germ cell tumours. This retrospective analysis shows a survival benefit for patients treated with HDCT as an intensification of first salvage treatment, but not for low-risk patients, for whom no significant differences in Overall Survival (OS) were observed in the two cohorts.^44^ Prospective studies are ongoing to further investigate this.

CDCT salvage treatments include regimens with paclitaxel, ifosfamide and cisplatin (TIP) or vinblastine, ifosfamide, and cisplatin (VeIP), while other regimens play a marginal role (gemcitabine and oxaliplatin, gemcitabine, irinotecan combined with nedaplatin, combinations with taxanes). On the other hand, the main regimens used for HDCT comprise of carboplatin and etoposide, followed by autologous stem cell transplant. Another effective HDCT regimen is TI-CE (paclitaxel plus ifosfamide, followed by high-dose carboplatin plus etoposide, which has proven its efficacy in patients affected by CC and other NSGCTs with poor prognostic features.^45^ HDCT plus ASCT should be taken into consideration for specific patients with resistant choriocarcinoma and it should be performed in tertiary referral centres, with adequate experience in the management of this treatment and its adverse events. The short-term toxicities of HDCT include gastrointestinal, hepatic, pulmonary, renal, and neurological effects. Long-term toxicities include leukaemia (related to the cumulative dose of etoposide, with doses >2 g/m² associated with a 2–3% risk of leukaemia), hearing loss, tinnitus, and peripheral neuropathy.^46^

The role of a maintenance therapy with oral etoposide after HDCT is debated. At the moment there is an ongoing phase I/II trial which is investigating maintenance zanzalintinib, a multi-tyrosin-kinase inhibitor, in combination with oral etoposide following high-dose chemotherapy in patients with relapsed metastatic germ cell tumours (presented at ASCO GU Symposium 2025).

When a patient is deemed ineligible for HDCT or the disease progresses after such treatment, investigation of novel therapeutic agents and inclusion in clinical trials is probably the best option. Indeed, third line schemes listed before, such as GemOX, can only be considered palliative, as the remission rate is very low.

The most frequent genomic alterations found in platinum-resistant GCTs are in the Ras and PI3K/AKT/mTOR pathways and in the p53-MDM2 axis.^47^ For this reason, the development of targeted therapies for refractory GCTs is ongoing, with a focus on these signal pathways. It should be underlined that the aim of new targeted agents in clinical trials is to gain a longer response with manageable side effects with the endpoint of improving overall survival, but not with the aim of complete remission. Among the PI3K/AKT/mTOR pathway activators, there are several tyrosine kinases which are implied in GCTs pathogenesis, including KIT, ERBB2, PDGFR and VEGFR.^48^

While some tyrosine kinases inhibitors (TKIs) which are effective for different oncological diseases were shown to inhibit tumour growth in GCT models *in vitro*, clinical therapeutic response to TKIs such as sunitinib, pazopanib, imatinib, cabozantinib and everolimus have been limited to case reports.^49^

As VEGF receptors are overexpressed in GCTs, some Authors hypothesised that a combination of hemotherapy and bevacizumab could improve the outcomes in refractory GCTs, similarly to what happens in ovarian cancer. However, no improvement in the survival or progression-free survival outcomes was detected in clinical trials with bevacizumab.^50^

As other platinum sensitive neoplasms, a sensitivity of GCTs to poly (ADP-ribose) polymerase (PARP) inhibitors has also been hypothesised. PARP inhibitors work with a mechanism called synthetic lethality, inhibiting DNA repair mechanisms in already DNA repair deficient tumour cells, for instance in BRCA mutated tumours. In a clinical trial which enrolled 124 patients with GCTs, the expression rate of PARP in tumour tissue was demonstrated to be high in many cases, in particular 52.6% in seminomas, 47% in embryonic carcinomas, 33.3% in yolk sac tumours, 26.7% in teratomas and 25% in choriocarcinoma. The aim of this study was to determine differences in OS according to PARP expression. Patients with low PARP expression in tumour tissue showed a trend for better OS but the difference was not statistically significant. PARPi Olaparib has been tested in a clinical trial as a single agent in patients with refectory GTs, but unfortunately it demonstrated only limited activity, the best result being a four-month radiographic stability in a single patient harbouring BRCA mutation.^51^

Another possible target for therapies is CD30 cell surface protein. This receptor is usually expressed in different kinds of lymphoma and embryonal carcinoma. However, it can also be found on seminoma cells, both in the pure seminoma histology or in the seminomatous part of mixed GCTs. The presence of CD30 seems to be a marker of worse prognosis. Indeed patients with GCT cells expressing high levels of CD30 showed worse progression-free survival (PFS) and OS compared to patients with CD30-negative tumours. Brentuximab-vedotin, an anti-CD30 antibody drug conjugate, showed efficacy in correlation with CD30 expression in TGCT cell lines. Therefore, CD30-positive TGCTs may benefit from brentuximab vedotin therapy in a clinical setting. Two clinical trials^52,53^ have tested in a limited number of patients the potential benefit of this treatment in relapsed TGCTs, already treated with traditional platinum-based regimens. However, only a small proportion of patients in those trials showed a tumour response.^54^

As immunotherapies have demonstrated to improve survival in many cancer types in recent years, these therapies have been tested also for GCTs. There are two main trials which explored the role of PD-L1 as a biomarker in the treatment of GCTs. Both studies reported a higher expression of PD-L1 in tumour tissue when compared with normal testis, and patients with GCT with a low PD-L1 expression had significantly improved PFS and OS compared with patients with high PD-L1 expression. In particular, choriocarcinoma exhibits the highest level of PD-L1 (52.6%). In those studies, high PD-L1 expression was associated with poor prognostic features, such as more than three metastatic sites, non-pulmonary visceral metastases and elevated serum tumour markers.^55^ Additionally, it was found that cytotoxic T-lymphocyte-associated antigen 4 (CTLA-4) was significantly expressed in yolk sac tumours, while PD-L1 tumour cell positivity was significantly more frequent in choriocarcinomas. Indeed, choriocarcinoma is the only subtype of GCTs that expresses PD-L1 on tumour cells, while other subtypes express PD-L1 primarily on tumour-associated macrophages.^56^

The first study of immunotherapy efficacy in GCTS was a phase II trial investigating pembrolizumab in patients with no curable options. The trial confirmed that Pembrolizumab is well tolerated even in this setting, but it does not have any clinically meaningful activity.^57^

Another phase II trial evaluated the efficacy and safety of nivolumab for relapsed GCTs^58^ in a cohort of 17 adults. The trial included PD-L1 assessment and genomic sequencing. Nivolumab did not show meaningful clinical activity, with only one patient showing a durable partial response, associated with a high tumour mutational burden.

In summary, the use of anti-PD1 does not seem to improve outcomes for patients with refractory GCTs. The low rate of somatic mutations resulting in the absence of potential neoantigens, which are crucial for T-cell response, may explain the unimpressive results of immunotherapy in the treatment of GCTs.

## Conclusion

Choriocarcinoma is a rare subtype of testicular neoplasm with highly heterogeneous behaviour. It is predominantly aggressive and associated with a poor prognosis, especially in cases with visceral and brain metastases. For metastatic patients, despite the timely initiation of chemotherapy, the high tumour burden and rapid disease progression often lead to unfavourable outcomes.

In addition to these characteristics, there is the risk of choriocarcinoma syndrome, a rare but life-threatening complication for which the optimal therapeutic approach remains to be defined and which requires early detection, prompt referral to a tertiary centre and adequate systemic therapy.

In conclusion, within the context of GCTs with a general good prognosis even when metastatic, choriocarcinoma is a rare subtype that needs to be identified and managed effectively in order to offer patients the best treatment options and ensure the best survival rate.
